# Genetic variation of *Amaranthus retroflexus* L. and *Chenopodium album* L. (Amaranthaceae) suggests multiple independent introductions into Iran

**DOI:** 10.3389/fpls.2022.1024555

**Published:** 2023-01-04

**Authors:** Shiva Hamidzadeh Moghadam, Mohammad Taghi Alebrahim, Mehdi Mohebodini, Dana R. MacGregor

**Affiliations:** ^1^ Department of Plant Production and Genetics, University of Mohaghegh Ardabili, Ardabil, Iran; ^2^ Department of Horticultural Sciences, University of Mohaghegh Ardabili, Ardabil, Iran; ^3^ Protecting Crops and the Environment, Rothamsted Research, Harpenden, United Kingdom

**Keywords:** biogeography, population diversity, genetic variability, weedy plants, ISSR markers

## Abstract

*Amaranthus retroflexus* L. and *Chenopodium album* L. (Amaranthaceae) are weedy plants that cause severe ecological and economic damage. In this study, we collected DNA from three different countries and assessed genetic diversity using inter-simple sequence repeat (ISSR) markers. Our analysis shows both weed species have low genetic diversity within a population and high genetic diversity among populations, as well as a low value of gene flow among the populations. UPGMA clustering and principal coordinate analysis indicate four distinct groups for *A. retroflexus* L. and *C. album* L. exist. We detected significant isolation-by-distance for *A. retroflexus* L. and no significant correlation for *C.*album L. These conclusions are based data from 13 ISSR primers where the average percentage of polymorphism produced was 98.46% for *A. retroflexus* L. and 74.81% for *C. album* L.These data suggest that each population was independently introduced to the location from which it was sampled and these noxious weeds come armed with considerable genetic variability giving them the opportunity to manifest myriad traits that could be used to avoid management practices. Our results, albeit not definitive about this issue, do not support the native status of *C. album* L. in Iran.

## 1 Introduction

Evolutionary genetics tools are valuable for revealing the genetic diversity within and between populations. Within the field of Weed Science, these tools have been applied to understanding the traits or genes that facilitate successful establishment by weedy species (Sakai et al., 2001; Lee, 2002; Majd et al., 2020). Factors that facilitate rapid and efficient colonization of new habitats include: wide environmental tolerance, phenotypic flexibility, inbreeding coefficient or ability to undergo asexual reproduction, efficient dispersal abilities, high relative growth rate, and high ability to compete (te Beest et al., 2012). The Amaranthaceae weeds redroot pigweed (*Amaranthus retroflexus* L.) and lamb’s quarters (*Chenopodium album* L.) are problematic cosmopolitan weeds that inhabit a wide variety of habitats across the globe (Horak and Loughin, 2000; CABI, 2020; Khan et al., 2022; Tang et al., 2022). Even when grown in common garden conditions they exhibit significant morphological and biochemical diversity in reproductive and metabolic traits that are important for successful establishment and survival in new locations (Alebrahim et al., 2012; Hamidzadeh et al., 2021). They are predominantly self-pollinating (Kulakow and Hauptli, 1994; Eslami and Ward, 2021) and have vigorous and highly adapted reproductive traits which maximize their ability to generate and maintain seed banks (Toole and Brown, 1946; Maurya and Ambasht, 1973; Holm et al., 1977; Knezevic and Horak, 1998; Telewski and Zeevaart, 2002; CABI, 2020). Moreover, these fast-growing and highly competitive annual plants cause large yield losses across much of the world’s agricultural areas (Horak and Loughin, 2000; CABI, 2020). These two weeds are therefore highly successful bioinvaders that need to be understood and managed.

These two weeds are also good systems for investigating the genetic fingerprints of weediness and weedy traits. Although it is autogamous, *C. album* is highly polymorphic, even compared to other species within the *Chenopodium* genus. Previous studies explored this taxonomic complexity through cytology (Mukherjee, 1986), karyotypic analysis (Kolano et al., 2008), flavonoid profiling (Rahiminejad and Gornall, 2004) random amplified polymorphic DNA profiles (Rana et al., 2010), ISSR marker analysis (Rana et al., 2012) and cpDNA regions sequencing (Mandák et al., 2018; Krak et al., 2019). *A. retroflexus* is partly autogamous and a study of the genetic composition of Central European *A. retroflexus* using isoenzyme analysis showed moderate levels of genetic diversity and strong evidence for inbreeding within populations compared to other herbaceous plants (Mandák et al., 2011). Therefore, there is a precedence for using *A. retroflexus* and *C. album* for evolutionary genetic studies. Despite this precedence, little is known about the genetic diversity of these species within and between populations in places where they have successfully established as weeds.

The objective of this study was to characterize the genetic diversity of Iranian, French and Spanish *A. retroflexus* L. and *C. album* L. populations that are known to exhibit diversity in several important morphological and biochemical traits (Alebrahim et al., 2012; Hamidzadeh et al., 2021). Regarding these populations, we hypothesized that (1) individuals from multiple different founder sources gave rise to the Iranian, French and Spanish populations of *A. retroflexus* L. and *C. album* L. that can be found at the sample locations, and (2) geographic distance and resistance of gene flow to altitudinal differences drive population genetic differentiation, i.e. isolation-by-distance (IBD), both of which would manifest as higher levels of genetic diversity when grown in common garden.

## 2 Materials and methods

### 2.1 Plant materials

Seeds of 16 A*. retroflexus* and 17 C*. album* populations were collected in 2016 and 2017 from different provinces of Iran, Spain, and France (**Table 1** and **Supplemental Figures 1A, B**). Further details regarding how these specific samples were collected as well as detailed characterisation and analysis of morphological and biochemical traits can be found at Hamidzadeh et al. (2021). The seeds provided by Research Institute of Forests and Rangelands (RIFR) and UMR Agroecology (INRA Dijon) were cultivated at the experimental field of the agriculture research of University of Mohaghegh Ardabili (38˚ 19N 48˚ 20E). Three weeks after sowing, five plants per population were selected and planted outdoors at the experimental field of the agriculture research of University of Mohaghegh Ardabili during the summer of 2018. Three replicated plots with five seedlings per replicate were planted in each plot. Seedlings were planted at a distance of 20 cm in row and 30 cm between rows (Hamidzadeh et al., 2021). For DNA extraction fresh leaves were taken from individual plants of each genotype of two weeks old seedlings. The leaf samples were preserved at − 80°C until the DNA extraction was executed.

**Table 1:** The list of 16 A*. retroflexus* and 17 C*. album* populations evaluated in this study with their coordinate and origin names.

***A. retroflexus***			
No.	Region name	Origin name	Latitude (N)	Longitude (E)
1	Rasht	Iran	37°16'05 N	49°35'20 E
2	Gorgan	Iran	36°45'06 N	54°21'40 E
3	Rudsar	Iran	37°08'16 N	50°17'10 E
4	Sari	Iran	36°33'57 N	53°03'31 E
5	Shahr-e-Rey	Iran	35°34'37 N	51°27'44 E
6	Ilam	Iran	33°38'05N	46°24'54 E
7	Yazd	Iran	31°10'97 N	53°11'97 E
8	Bojnurd	Iran	37°53'74 N	57°24'96 E
9	Zarand	Iran	30°47'27 N	56°50'10 E
10	Hamedan	Iran	34°47'50 N	48°30'45 E
11	Ardabil	Iran	38°14'54 N	48°17'03 E
12	Moghan	Iran	39°13' 00 N	47°33'53 E
13	France	France	47°19'20 N	5°2'28 E
14	Spain 1	Spain	37°53'18 N	4°46'38 W
15	Spain 2	Spain	37° 53' 15 N	4° 46'35 W
16	Spain 3	Spain	37° 53' 14 N	4° 46'45 W
***C. album***			
1	Rudsar	Iran	37°08'13 N	50°16'52 E
2	Rasht	Iran	37°16'03 N	49°35'08 E
3	Boyer-Ahmad	Iran	30°53'47 N	51°24'96 E
4	Rudan	Iran	27°25'44 N	57°10'45 E
5	Moghan	Iran	39°12'03 N	47°34'24 E
6	Kivi	Iran	37'41'02 N	48°20'53 E
7	Ardabil	Iran	38°12'44 N	48°17'38 E
8	Yazdabad	Iran	32°39'41 N	51°41'21 E
9	Shahr-e-Ray	Iran	35°34'22 N	51°27' 44 E
10	Tehran	Iran	35°41'13 N	51°26'22 E
11	Dehloran	Iran	32°41'49 N	47°16'05 E
12	Hamadan	Iran	34°49'46 N	48°19' 47 E
13	Mashhad	Iran	36°16'24 N	59°38'16 E
14	Spain 1	Spain	37° 53' 15 N	4° 46'35 W
15	Spain 2	Spain	37° 53' 14 N	4° 46'45 W
16	France 1	France	47°19'20 N	5°2'28 E
17	France 2	France	47°19'29 N	5°2'22 E

### 2.2 DNA extraction and ISSR analysis

To test hypotheses, we used inter simple sequence repeat (ISSR) markers. ISSR markers are highly reproducible and accurate tools that generate highly reproducible banding patterns from a single polymerase chain reaction (PCR) amplification (Raut et al., 2014; Stefunova et al., 2014). Although newer technique are available, ISRR makers have historically (Wolfe et al., 1998) and recently (Yan et al, 2019; Kwiecińska-Poppe et al., 2020; Tang and Ma, 2020; Liu et al., 2021; Alotaibi and Abd-Elgawad, 2022; Flihi et al., 2022; Ghanbari et al., 2022; Haq et al., 2022) been used successfully for diversity studies and structuring of natural populations.

Genomic DNA was isolated from the young leaves of plants according to the cetyltrimethylammonium bromide (CTAB) method described by Saghai-Maroof et al. (1984). The DNA concentration and purity were determined with a Thermo™ Scientific NanoDrop™ spectrophotometer and visually verified *via* 0.8% (w/v) agarose gel electrophoresis. 52 ISSR primers (synthesized by CinnaGen Co., Teheran, Iran) from the University of British Columbia’s UBC set no. 9 (Vancouver, British Columbia, Canada) were screened for PCR amplification and thirteen primers that produced clear, reproducible banding patterns were chosen (**Table 2**). We compensated for potential pitfalls in the use of ISRR markers (such as sensitivity to the quality and concentration of template DNA, concentrations of PCR components, PCR cycling conditions as well as electrophoretic conditions).

**Table 2:** Data of ISSR primers of 13 primers in *A. retroflexus* **(A)** and *C. album* **(B)** populations.

**A)**															
Primer name	Primer seq	Tm	NT	NP	PP	β	PIC	EMR	MI	RP	MRP	Na	Ne	H	I
AL-1	(GA)6CC	43.7	6	6	100	1	0.378	6	2.26	3.25	19.5	2	1.63	0.4	0.55
AL-2	GA(GGA)2GGC	38	5	4	80	0.8	0.345	3.2	1.1	2.625	10.5	1.8	1.44	0.34	0.49
UBC839	(AC)8GA	53	4	4	100	1	0.449	4	1.79	3	12	2	1.81	0.44	0.63
UBC810	(GA)8T	52	13	13	100	1	0.401	13	5.21	7.87	102.31	2	1.71	0.37	0.58
UBC834	(AG)8YT	54	4	4	100	1	0.549	4	2.19	2.25	9	2	1.62	0.36	0.54
UBC829	(TG)8C	49	3	3	100	1	0.445	3	1.335	2.125	6.375	2	1.81	0.44	0.63
UBC818	(CA)8G	42	4	4	100	1	0.449	4	1.796	3	12	2	1.82	0.44	0.63
UBC822	(TC)8A	49	3	3	100	1	0.401	3	1.203	1.87	5.61	2	1.73	0.39	0.57
UBC811	(GA)8C	52.4	4	4	100	1	0.395	4	1.58	2.5	10	2	1.68	0.39	0.57
UBC819	(GT)8A	52.4	3	3	100	1	0.466	3	1.398	2.37	7.11	2	1.87	0.46	0.65
UBC815	(CT)8G	52	4	4	100	1	0.465	4	1.86	3	12	2	1.86	0.45	0.65
UBC833	(AT)8YG	54	3	3	100	1	0.495	3	1.485	2.75	8.25	2	1.97	0.49	0.68
UBC817	(CA)8A	49	3	3	100	1	0.458	3	1.374	2.5	7.5	2	1.86	0.45	0.64
Mean	–	–	4.54	4.46	98.46	0.98	0.44	4.4	1.89	3.01	17.09	1.98	1.75	0.42	0.6
**B)**															
Primer name	Primer seq	Tm	NT	NP	PP	β	PIC	EMR	MI	RP	MRP	Na	Ne	H	I
AL-1	(GA)6CC	43.7	4	3	75	0.75	0.327	2.25	0.736	2	6	1.75	1.54	0.29	0.43
AL-2	GA(GGA)2GGC	38	3	1	33.33	0.33	0.162	0.33	0.053	0.35	0.35	1.33	1.13	0.09	0.15
UBC839	(AC)8GA	53	3	3	100	1	0.453	3	1.359	2.47	7.41	2	1.89	0.46	0.66
UBC810	(GA)8T	52	8	7	87.5	0.87	0.304	6.12	1.860	4.59	32.13	1.87	1.64	0.35	0.52
UBC834	(AG)8YT	54	5	3	60	0.6	0.09	1.8	0.162	0.94	2.82	1.6	1.21	0.156	0.25
UBC829	(TG)8C	49	3	3	100	1	0.266	3	0.798	1.76	5.28	2	1.68	0.39	0.58
UBC818	(CA)8G	42	3	3	100	1	0.463	3	1.389	2.24	6.72	2	1.82	0.44	0.63
UBC822	(TC)8A	49	3	2	66.66	0.66	0.267	1.33	0.355	1.06	2.12	1.66	1.41	0.24	0.6
UBC811	(GA)8C	52.4	3	1	33.33	0.33	0.161	0.33	0.053	0.71	0.71	1.3	1.28	0.15	0.21
UBC819	(GT)8A	52.4	3	2	66.66	0.66	0.24	1.33	0.319	0.94	1.88	1.66	1.37	0.23	0.36
UBC815	(CT)8G	52	4	4	100	1	0.362	4	1.448	3.06	12.24	2	1.88	0.46	0.65
UBC833	(AT)8YG	54	4	2	50	0.5	0.237	1	0.237	0.82	1.64	1.5	1.24	0.15	0.24
UBC817	(CA)8A	49	3	3	100	1	0.481	3	1.443	2	6	2	1.77	0.42	0.61
Mean	–	–	3.77	2.85	74.81	0.75	0.29	2.35	0.79	1.76	6.56	1.74	1.53	0.29	0.45

melting temperature (Tm), number of total bands (NT), number of polymorphic bands (NP), percentage of polymorphic fragment (PP), polymorphic information content (PIC), effective multiplex ratio (EMR), marker index (MI), resolving power (RP), mass resolving power (MRP), number of observed alleles (Na), number of effective alleles (Ne), Nei’s gene diversity (H), Shannon’s information index (I).

melting temperature (Tm), number of total bands (NT), number of polymorphic bands (NP), percentage of polymorphic fragment (PP), polymorphic information content (PIC), effective multiplex ratio (EMR), marker index (MI), resolving power (RP), mass resolving power (MRP), number of observed alleles (Na), number of effective alleles (Ne), Nei’s gene diversity (H), Shannon’s information index (I).

Bio-Rad T100™ thermal cycler (Bio-Rad Laboratories, Inc. Hercules, CA, USA) started with 4 min at 94°C, and 40 cycles of 1 min at 94°C, 75 s at each primer’s annealing temperature (**Table 2**) and 2 min at 72°C ended by an extension for 10 min at 72°C. The PCR products were separated on 2% agarose gel in 1X TBE buffer then ran at 90 voltage for 1 hours, stained with DNA-safe stain (CinnaGen, Iran) and photographed with a digital imaging system (UV tech, Germany). Molecular weights were estimated using 50 bp DNA Ladder (CinnaGen, Iran). An example of the banding pattern observed in shown in **Supplemental Figure 2**.

### 2.3 Data analysis

Among the 52 primers tested, 13 produced clearly and reproducibly amplified ISSR fragments. These were scored based on a binary matrix for presence (1) or absence (0) of bands. Discriminatory power of the primers was evaluated by means of resolving power (Rp), mass resolving power (MRP), polymorphic information content (PIC), marker index (MI). Rp of each primer which is the ability of each primer to detect level of variation between individuals was calculated according to (Prevost and Wilkinson, 1999):


[1] $$\text{Rp}=\sum^{}\text{bI}$$

where bI (band informativeness) takes the values of: 1–[2|0.5–p|], where p is the proportion of individuals containing the band. Further, mean resolving power (MRP) for each primer was calculated via


[2] $$\text{MRP}=\;\frac{1}{\text{n }}\;\sum^{}\text{bI}$$

following (Milbourne et al., 1997). PIC value was calculated according to (Roldán-Ruiz et al., 2000):


[3] PIC=2fi(1−fi)

where fi is the frequency of fragments present in that locus and (1−fi) is the frequency of the null allele. MI, a measure of overall utility of a molecular marker technique, for each primer was calculated as a product of two functions, the polymorphic information content and effective multiplex ratio (EMR) (Milbourne et al., 1997), i.e.,


[4] MI=PIC×EMR

The effective multiple ratio (EMR=npβ ) is the product of the number of polymorphic loci (np) in the population analyzed and the fraction of markers that were polymorphic (β) (Powell et al., 1996).

The binary data matrix was analyzed using POPGENE version 1.32 (Yeh and Boyle, 1997) to examine different genetic diversity parameters including number of polymorphic loci (PL), percentage of polymorphic loci (PPL), Observed number of alleles (Na), Effective number of alleles (Ne), Nei’s gene diversity (H), Shannon’s information index (I(. At the species wide level, total genetic diversity (Ht), genetic diversity within populations (Hs) and Nei (1973) coefficient of genetic differentiation among populations calculated via


[5] Gst=(Ht−Hs)/Ht

Corresponding estimates of gene flow (Nm), i.e. the average per generation number of migrants exchanged among populations, was calculated based on (McDermott and McDonald, 1993):


[6] Nm=0.5(1−GST)/GST

To examine the genetic relationship among populations, unbiased genetic distance and genetic identity (Nei, 1978) were also calculated by POPGENE and a dendrogram was constructed from Nei’s genetic distance with the unweighted pair-group method of averages (UPGMA) using NTSYSpc 1.02 software (Rohlf, 2000). To determine the quality of clustering (Saracli et al., 2013), Bootstrapped cluster analysis (UPGMA) was used to measure cophentic correlation coefficient (r) based on (Rohlf and Sokal, 1981). Principle coordinate analysis (PCoA) to assess genetic diversity were also calculated (Mohammadi and Prasanna, 2003). To evaluate genetic variance, analysis of molecular variance (AMOVA) (Excoffier et al., 1992) was carried out using GenAlEx version 6.4. From AMOVA, the fixation index (Fst) were obtained (Peakall and Smouse, 2006). To determine whether weedy population genetic structure followed a pattern of isolation by distance, genetic distance matrices were correlated with geographical distance matrices using a Mantel test in GenAlEx.

## 3 Results

### 3.1 Statistics of DNA marker used in genetic diversity assessment

**Table 2** indicates that the ISSR primers used herein accurately and sufficiently measure the degree of polymorphism present in the populations and are sufficiently powerful to differentiate between populations; therefore, they were suitable for assessing genetic diversity of these populations. The level of polymorphism revealed by the ISSR approach was very high and reached 98.46% for *A. retroflexus* L. and 74.81% for *C. album* L. within analyzed materials. These differentiating loci are therefore suitable for evaluating the genetic variability of these populations. Moreover based on PIC values, it can be concluded that the capacity of the marker system to detect polymorphic loci in a single amplification was very efficient; the average value of this coefficient amounted 0.78 for *A. retroflexus* L. and 0.71 for *C. album* L.. These results demonstrate this technique can be conveniently used for the genetic characterization of these populations of *A. retroflexus* L. and *C. album* L. Use of ISSR markers are also recently reported as a functional markers elsewhere (Sivaprakash et al., 2004; Yan et al., 2019; Kwiecińska-Poppe et al, 2020; Alotaibi and Abd-Elgawad, 2022; Flihi et al., 2022; Ghanbari et al., 2022; Haq et al, 2022).

***A. retroflexus*:** Against our *A. retroflexus* L. DNA, the 13 ISSR primers produced a total of 59 bands, of which 58 were polymorphic. The number of polymorphic bands ranged from 3 (UBC822, UBC829, UBC819, UBC833 and UBC817) to 13 (UBC810). The ISSR pattern obtained with UBC810 primer is demonstrated in **Supplemental Figure 2A**. The Al2 primer generated the minimum polymorphism of 80% and primers AL1, UBC839, UBC810, UBC834, UBC829, UBC818, UBC822, UBC811, UBC819, UBC815, UC833 and UC817 showed 100% polymorphism. While the highest Rp and MRP value was recorded at 7.87 and 102.31 (UBC810), the lowest was at 1.87 and 5.61 (UBC822), respectively. The EMR was the highest for UBC810 (13) and lowest for UBC822, UBC829, UBC819, UBC833 and UBC817 (3). Similarly, marker index (MI) value was highest for UBC810 (5.21) and lowest for AL2 primer with 1.1. The observed number of alleles (Na) was recorded low for the primer AL2 (1.8). The effective number of allele (Ne) was invariably less than Na values showing a variation in the range of 1.44 (AL2) to 1.97 (UBC833). The Shannon index (I) estimates were low, ranging from 0.49 (AL2) to 0.68 (UBC833), as well as the estimates of Nei’s genetic diversity (H), ranging from 0.34 (AL2) to 0.49 (UBC833) (**Table 2A**).

***C. album***: These 13 selected primers generated 49 ISSR bands in the 17 C*. album* populations, 3 to 8 bands per primer, of which 37 were polymorphic. The number of polymorphic bands varied from 1 in Al2 and UBC811 to 7 in UBC810. The ISSR pattern obtained with UBC810 primer is demonstrated in **Supplemental Figure 2B**. Al2 and UBC811 also provided the minimum polymorphism of 33.33% and primers UBC839, UBC829, UBC818, UBC815 and UBC817 showed 100% polymorphism. The highest Rp and MRP value was in UBC810 primer (4.59 and 32.13 respectively), and the lowest one in AL2 (0.35 and 0.35 respectively). The EMR was the highest for UBC810 (6.12) and lowest for UBC839, UBC829, UBC818 and UBC817 (3). Similarly, marker index (MI) value was highest for UBC810 (1.86) and lowest for AL2 and UBBC811 primers with 0.053. AL2 and UBC811 have the lowest (1.3) observed number of alleles (Na) and UBC839, UBC829, UBC818, UBC815 and UBC817 (2) having the highest. The effective number of allele (Ne) was invariably less than Na values showing a variation in the range of 1.13 (AL2) to 1.89 (UBC839). The Shannon index (I) ranging from 0.15 (AL2) to 0.66 (UBC839), as well as the estimates of Nei’s genetic diversity (H), ranging from 0.09 (AL2) to 0.46 (UBC839 and UBC815) (**Table 2B**).

The PIC values ranged from 0.345 to 0.549 with the highest being for primer UBC834 and the lowest for primer AL2 for *A. retroflexus* L. (**Table 2A**). Furthermore, UBC834 primer with 0.09 and UBC817 primer with 0.48 showed the lowest and greatest PIC value among all primers for *C. album* populations, respectively (**Table 2B**). Our results showed that the PIC values gave an average PIC value of 0.44 for *A. retroflexus* L and 0.29 for *C. album* population, suggesting that all the markers fell within the moderately informative category defined by Botstein et al. (1980) for *A. retroflexus* L and moderately or low informative category for *C. album*.

### 3.2 Genetic diversity and population structure of *A. retroflexus* L. and *C. album* L.:

Genetic variability represents vital information about historic bottleneck effects and diversification since establishment and understanding a population’s history informs choices about which innovative weed control options would be most suitable (Goolsby et al., 2006; Slotta, 2008). Knowing what level of genetic variation exists within and between populations is therefore essential for developing strategic and effective weed control practices as different responses to chemical or biological control methods will be underpinned by differences in the weed genomes (Arias et al., 2011).

**Figure 1:**
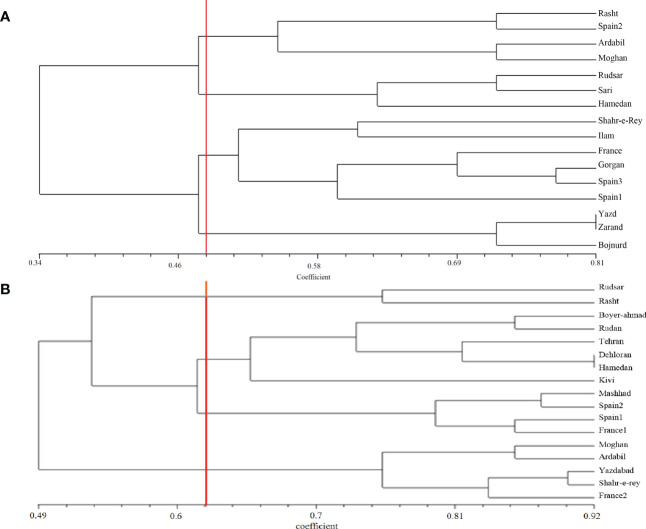
UPGMA clustering of *A retroflexus* **(A)** and *C album* **(B)** populations based on Jaccard similarity coefficient calculated from ISSR markers.

**Figure 2:**
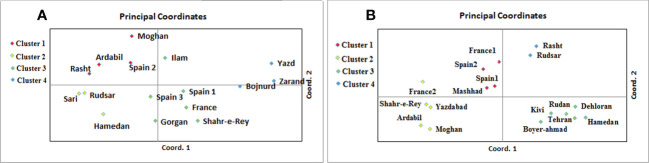
Principal coordinates analysis of 16 A*. retroflexus* **(A)** and 17 C*. album* **(B)** populations based on the genetic variation revealed by ISSR.

***A. retroflexus*:** The UPGMA clustering algorithm from ISSR analysis grouped the 16 A*. retroflexus* L. populations into four distinct clusters at a similarity index value of 0.46 (**Figure 1A**). The correlation cophenetic value (r) calculated by Mantel test (0.78) indicates a high grouping efficiency. However, these groups do not cluster based on geographic proximity, e.g. the Spanish populations fall across two separate groups and the Iranian populations are not clustered according to geographical distance. The first cluster consists of Rasht, Spain2, Ardabil and Moghan. The second group includes Rudsar, Sari and Hamedan populations. The third cluster is a representation of the populations from Shahre-e-Rey, Ilam, France, Gorgan, Spain1 and Spain3. The fourth group was formed of Yazd, Zarand and Bojnurd. Analysis of molecular variance confirmed the cutoff point of clustering (phipt=0.21) (**Table 3A**). Confirming the results of the UPGMA clustering, Principal Coordinates Analysis (PCoA) also showed four main clusters (**Figure 2A**).

**Table 3:** Analysis of Molecular Variance (AMOVA) for *A. retroflexus* **(A)** and *C. album* **(B)** populations.

**A)**					
Source	df	Sum of squars	Variance components	Percentage of variation	PhiPT
Among populations	1	35.967	35.967	81	0.21^**^
Within populations	14	160.095	11.435	19	–
Total	15	196.063	–	100	–
**B)**					
Source	df	Sum of squars	Variance components	Percentage of variation	PhiPT
Among populations	1	27.769	27.769	78	0.31^**^
Within populations	15	98.467	6.564	22	–
Total	16	126.235	–	100	–

AMOVA (**Table 3A**) demonstrated strongly significant genetic differentiation among populations and within populations (P < 0.001); 81.0% of the total variation was due to differences among populations, while the remaining 19.0% was attributed to within-population differences. The measurements of genetic diversity are summarized in **Table 4A**. The number of observed alleles and number of effective alleles ranged between 1.152-1.254 (Ilam or Yazd to Ardabil) and 1.092–1.144 (Ilam or Yazd to Ardabil), respectively. The value of Nei’s gene diversity ranged from 0.055 to 0.089 with the highest for Ardabil population and the lowest for Ilam and Yazd population among the 16 populations. The average of Shannon’s Information Index for the 16 populations is 0.11 which again the maximum and the minimum are respectively belonging to Ardail, Ilam or Yazd populations. The highest number of polymorphic loci (PL) and percentage of polymorphic loci (PPL) both belong to Ardail while the lowest, belongs to Ilam and Yazd. The values for total species diversity for among population (HT), within population diversity (Hs) and mean coefficient of gene differentiation (GST) were 0.429, 0.073 and 0.829, respectively. The highest genetic identity is between Yazd and Zarand (0.79) which exhibit the lowest genetic distance (0.22). The maximum genetic distance is between Rasht and Zarand, moreover between Rasht and Yazd (1.08), which show the minimum genetic identity of 0.33 (**Table 5A**).Furthermore, the level of gene flow (Nm) was estimated to be 0.102 individual per generation between populations, suggesting that genetic exchange between populations was low.

**Table 4:** Genetic diversity data of 16 *A. retroflexus* **(A)** and 17 *C. album* **(B)** populations.

**A)**											
population	Na	Ne	H	I	PL	PPL	Ht	Hs	Gst	Nm	Fst
Rasht	1.203	1.131	0.076	0.114	12	20.34					
Gorgan	1.186	1.106	0.065	0.099	11	18.64					
Rudsar	1.203	1.122	0.073	0.11	12	20.34					
Sari	1.22	1.138	0.082	0.122	13	22.03					
Shahr-e- Rey	1.169	1.108	0.063	0.094	10	16.95					
Ilam	1.152	1.092	0.055	0.083	9	15.25					
Yazd	1.152	1.092	0.055	0.083	9	15.25					
Bojnurd	1.203	1.131	0.076	0.114	12	20.34					
Zarand	1.186	1.133	0.075	0.109	11	18.64					
Hamedan	1.237	1.137	0.083	0.127	14	23.73					
Ardabil	1.254	1.144	0.089	0.135	15	25.42					
Moghan	1.22	1.112	0.072	0.112	13	22.03					
France	1.203	1.113	0.07	0.107	12	20.34					
Spain 1	1.203	1.113	0.076	0.114	12	20.34					
Spain 2	1.22	1.138	0.082	0.122	13	22.03					
Spain 3	1.203	1.113	0.07	0.107	12	20.34					
Mean	1.201	1.12	0.073	0.11	–	–					
Total	2	1.784	0.429	0.616	59	100	0.429	0.073	0.829	0.102	0.71
**B)**											
population	Na	Ne	H	I	PL	PPL	Ht	Hs	Gst	Nm	Fst
Rudsar	1.142	1.093	0.054	0.08	7	14.29					
Rasht	1.142	1.104	0.058	0.084	7	14.29					
Boyer- Ahmad	1.163	1.123	0.068	0.098	8	16.33					
Rudan	1.142	1.093	0.054	0.08	7	14.29					
Moghan	1.163	1.123	0.068	0.098	8	16.33					
Kivi	1.183	1.132	0.074	0.108	9	18.37					
Ardabil	1.183	1.132	0.074	0.108	9	18.37					
Yazdabad	1.183	1.153	0.082	0.116	9	18.37					
Shahr-e- Ray	1.183	1.142	0.078	0.112	9	18.37					
Tehran	1.183	1.11	0.066	0.1	9	18.37					
Dehloran	1.163	1.112	0.064	0.09	8	16.33					
Hamadan	1.163	1.102	0.065	0.09	8	16.33					
Mashhad	1.142	1.104	0.058	0.084	7	14.29					
Spain 1	1.122	1.095	0.052	0.075	6	12.24					
Spain 2	1.163	1.112	0.064	0.094	8	16.33					
France 1	1.63	1.123	0.068	0.098	8	16.33					
France 2	1.163	1.104	0.058	0.084	7	14.29					
Mean	1.189	1.115	0.065	0.094	–	–					
Total	1.959	1.636	0.36	0.531	47	95.92	0.36	0.064	0.82	0.109	0.7

number of observed alleles (Na), number of effective alleles (Ne), Nei’s gene diversity (H), Shannon’s information index (I), number of polymorphic loci (PL), percentage of polymorphic loci (PPL), total population diversity for within population (Hs), among population diversity (Ht), coefficient of gene differentiation (Gst), gene flow (Nm), fixation index (Fst).

**Table 5:** Nei’s unbiased measures of genetic identity (above diagonal) and genetic distance (below diagonal) primers in *A. retroflexus* **(A)** and *C.album* **(B)**.

**A)**																
pop	Rasht	Gorgan	Rudsar	Sari	Ray	Ilam	Yazd	Bojnurd	Zarand	Hamedan	Ardabil	Moghan	France	Spain 1	Spain 2	Spain 3
**Rasht**	1	0.72	0.71	0.64	0.5	0.49	0.33	0.47	0.33	0.59	0.71	0.55	0.47	0.47	0.59	0.59
**Gorgan**	0.31	1	0.71	0.61	0.61	0.42	0.37	0.5	0.44	0.52	0.67	0.52	0.54	0.47	0.62	0.62
**Rudsar**	0.33	0.33	1	0.72	0.45	0.47	0.35	0.45	0.38	0.61	0.66	0.5	0.52	0.49	0.61	0.54
**Sari**	0.44	0.49	0.31	1	0.45	0.61	0.42	0.49	0.38	0.67	0.66	0.61	0.55	0.49	0.54	0.61
**Rey**	0.67	0.49	0.78	0.78	1	0.61	0.49	0.62	0.59	0.54	0.49	0.4	0.66	0.49	0.67	0.61
**Ilam**	0.71	0.85	0.74	0.49	0.49	1	0.47	0.5	0.5	0.62	0.57	0.62	0.54	0.5	0.55	0.62
**Yazd**	1.08	0.98	1.03	0.85	0.71	0.74	1	0.76	0.79	0.47	0.38	0.5	0.52	0.52	0.47	0.5
**Bojnurd**	0.74	0.67	0.78	0.71	0.46	0.67	0.27	1	0.72	0.61	0.52	0.5	0.66	0.62	0.61	0.61
**Zarand**	1.08	0.81	0.94	0.94	0.52	0.67	0.22	0.31	1	0.37	0.35	0.44	0.52	0.45	0.47	0.5
**Hamedan**	0.52	0.64	0.49	0.38	0.61	0.46	0.74	0.49	0.98	1	0.64	0.62	0.61	0.54	0.55	0.59
**Ardabil**	0.33	0.38	0.41	0.41	0.71	0.55	0.94	0.64	1.03	0.44	1	0.74	0.55	0.52	0.64	0.74
**Moghan**	0.58	0.64	0.67	0.49	0.89	0.46	0.67	0.67	0.81	0.46	0.29	1	0.47	0.5	0.49	0.59
**France**	0.74	0.61	0.64	0.58	0.41	0.61	0.64	0.41	0.64	0.49	0.58	0.74	1	0.55	0.67	0.67
**Spain 1**	0.74	0.74	0.71	0.71	0.71	0.67	0.64	0.46	0.78	0.61	0.64	0.67	0.58	1	0.67	0.61
**Spain 2**	0.52	0.46	0.49	0.61	0.38	0.58	0.74	0.49	0.74	0.58	0.44	0.71	0.38	0.38	1	0.76
**Spain 3**	0.52	0.46	0.61	0.49	0.49	0.46	0.67	0.49	0.67	0.52	0.29	0.52	0.38	0.49	0.27	1
**B)**																
**pop**	**Rudsar**	**Rasht**	**Boyer**	**Rudan**	**Moghan**	**Kivi**	**Ardabil**	**Ray**	**Tehran**	**Dehloran**	**Hamadan**	**Mashhad**	**Spain 1**	**Spain 2**	**France1**	**France 2**
**Rudsar**	1	0.75	0.53	0.63	0.51	0.59	0.48	0.55	0.59	0.57	0.57	0.57	0.57	0.61	0.67	0.63
**Rasht**	0.28	1	0.61	0.67	0.51	0.55	0.48	0.55	0.63	0.65	0.61	0.65	0.69	0.77	0.79	0.63
**Boyer**	0.63	0.49	1	0.85	0.65	0.65	0.59	0.65	0.77	0.79	0.79	0.75	0.67	0.71	0.69	0.65
**Rudan**	0.45	0.39	0.15	1	0.63	0.67	0.53	0.59	0.79	0.73	0.77	0.65	0.61	0.69	0.63	0.55
**Moghan**	0.67	0.67	0.42	0.45	1	0.59	0.85	0.75	0.55	0.53	0.53	0.61	0.61	0.61	0.59	0.75
**Kivi**	0.52	0.59	0.42	0.39	0.52	1	0.61	0.55	0.75	0.69	0.69	0.65	0.65	0.61	0.67	0.55
**Ardabil**	0.71	0.71	0.52	0.63	0.15	0.49	1	0.77	0.57	0.55	0.55	0.63	0.71	0.63	0.61	0.77
**Yazdabad**	0.63	0.55	0.28	0.42	0.25	0.63	0.22	0.89	0.61	0.63	0.63	0.83	0.79	0.79	0.73	0.85
**Ray**	0.59	0.59	0.42	0.52	0.28	0.59	0.25	1	0.67	0.57	0.57	0.81	0.73	0.77	0.71	0.83
**Tehran**	0.52	0.45	0.25	0.22	0.59	0.28	0.55	0.39	1	0.81	0.85	0.69	0.69	0.65	0.67	0.55
**Dehloran**	0.55	0.42	0.22	0.3	0.63	0.36	0.59	0.55	0.2	1	0.91	0.75	0.83	0.67	0.73	0.61
**Hamadan**	0.55	0.49	0.22	0.25	0.63	0.36	0.59	0.55	0.15	0.08	1	0.71	0.75	0.63	0.69	0.53
**Mashhad**	0.55	0.42	0.28	0.42	0.49	0.42	0.45	0.2	0.36	0.28	0.33	1	0.83	0.87	0.81	0.77
**Spain 1**	0.55	0.36	0.39	0.49	0.49	0.42	0.33	0.3	0.36	0.17	0.28	0.17	1	0.79	0.85	0.77
**Spain 2**	0.49	0.25	0.33	0.36	0.49	0.49	0.45	0.25	0.42	0.39	0.45	0.13	0.22	1	0.85	0.77
**France 1**	0.39	0.22	0.36	0.45	0.52	0.39	0.49	0.33	0.39	0.3	0.36	0.2	0.15	0.15	1	0.83
**France 2**	0.45	0.45	0.42	0.59	0.28	0.59	0.25	0.17	0.59	0.49	0.63	0.25	0.25	0.25	0.17	1
Positive correlations are indicated in blue and negative correlations in red.															

***C. album*:** The UPGMA dendrogram from ISSR analysis at a similarity index value of 0.62 is shown in **Figure 1B**. Cophenetic coefficient (r) of 0.71 indicates high grouping efficiency. The populations were separated into four distinct clusters, which again mix proximal populations. Analysis of molecular variance confirmed the cut-off point of clustering (phipt=0.31) (**Table 3B**). The first cluster consists of Rudsar and Rasht. The second cluster groups Boyer-Ahmad, Rudan, Tehran, Dehloran, Hamedan and Kivi. The third cluster is Mashhad, Spain1, Spain2 and France1, while the fourth cluster is a representation of the populations from Moghan, Ardabil, Yazdabad, Shahr-e-Rey and France2. Like before, the PCoA analysis showed four main clusters confirming the results of the UPGMA clustering (**Figure 2B**).

AMOVA (**Table 3B**) was carried out considering the 17 populations studied, calculating the molecular variation attributable to differentiation among and within the populations (P < 0.001). The highest percentage of variation was found among the populations (78.0%) and in lower proportion, between populations (22.0%). The measurements of genetic diversity are summarized in **Table 4B**. The number of observed alleles and number of observed effective alleles ranged between 1.122-1.183 (Spain1 to Kivi, Ardail, Yazdabad, Shahre-Ray and Tehran) and 1.093–1.153 (Rudsar or Rudan to Yazdabad), respectively. The value of Nei’s gene diversity ranged from 0.052 to 0.82 with the highest for Yazdabad population and the lowest for Spain1 population among the 17 populations. The average of Shannon’s Information Index for the 17 populations is 0.094 which the maximum and the minimum are respectively belonging to Yazdabad- Spain1 populations. The highest number of polymorphic loci (PL) and percentage of polymorphic loci (PPL) both belong to Kivi, Ardail, Yazdabad, Shahre-Ray and Tehran while the lowest, belongs to Spain 1. The values for total species diversity for among population (HT), within population diversity (Hs) and mean coefficient of gene differentiation (GST) were 0.36, 0.064 and 0.82, respectively. Furthermore, the level of gene flow (Nm) was estimated to be 0.109 individuals per generation between populations, suggesting that gene exchange between populations was low. Hamedan and Dehloran populations showed the highest genetic identity (0.91) with having the lowest genetic distance (0.08). The maximum genetic distance (0.71) and the minimum genetic identity (0.48) are between Ardabil and Rudsar along with Ardabil and Rasht populations (**Table 5B**).

To determine if there were spatial patterns of genetic variation, we used a Mantel test (Diniz-Filho et al., 2013) to estimate the degree of correlation between the genetic data we obtained from the ISSR markers and geographical distances between the sampling locations.

**Figure 3:**
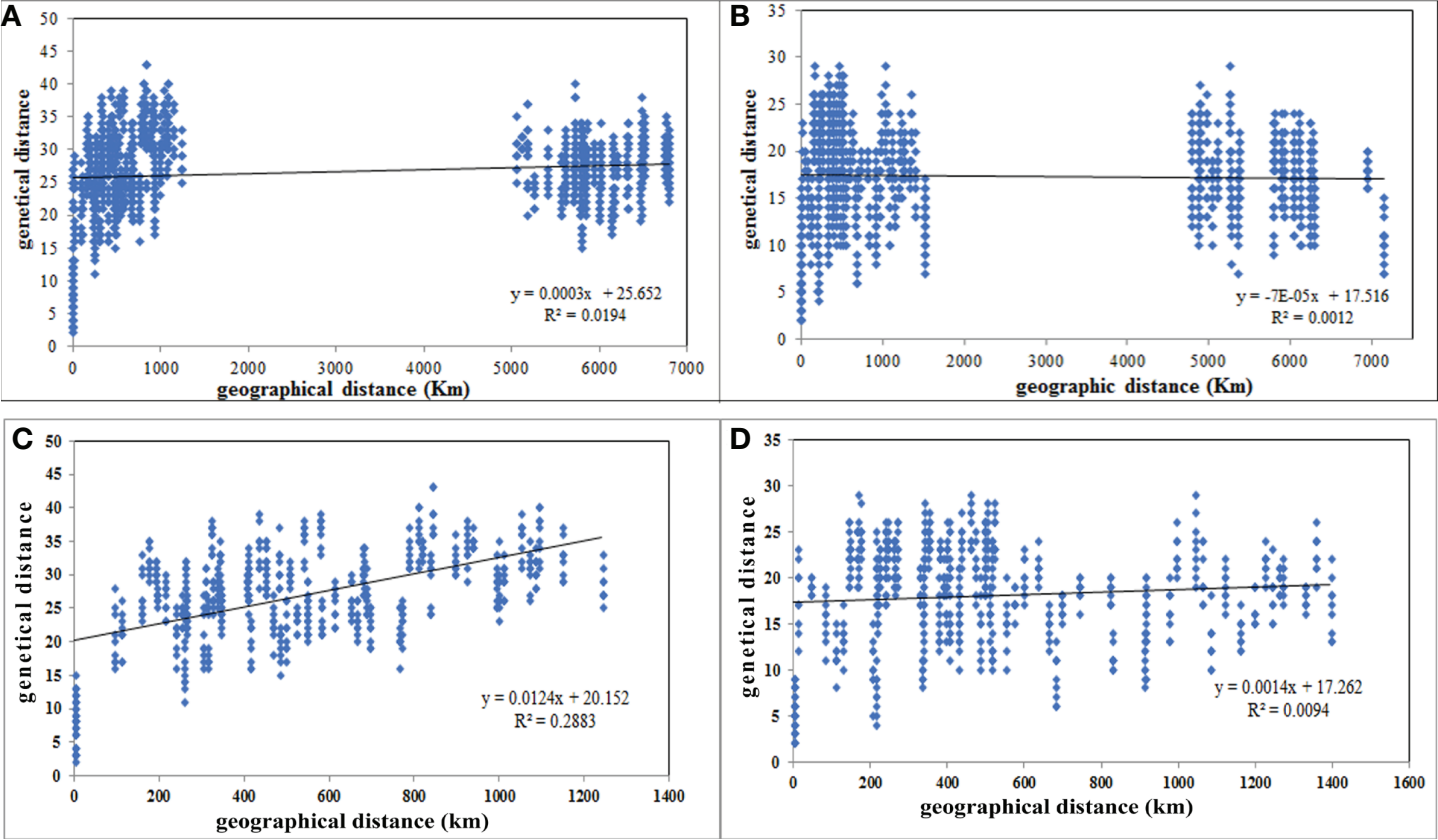
Scatterplot of pairwise genetic distance versus geographical distances (km) of 16 *A retroflexus* **(A)**, 17 C*. album* **(B)**, 12 iranian *A retroflexus* **(C)** and 13 C*. album* **(D)** populations based on “Isolation by Distance” analyses.

***A. retroflexus*:** Unlike the UPGMA clustering algorithm (**Figures 1**, **2**), which did not cluster groups based on geographic proximity, a significant correlation was detected between geographical distances and genetic distance for the 16 populations (r = 0.139, P (rxy-rand > = rxy-data) = 0.02) (**Figure 3A**), moreover, we observed a significant correlation for 12 Iranian populations (r = 0.537, P (rxy-rand > = rxy-data) = 0.01) (**Figure 3C**). The correlation plot for the 12 Iranian populations suggests a positive linear association between genetic and geographic distance, but the R^2^ value is very low. These analyses indicate that nearby populations tend to be genetically more similar to each other than expected by chance and there is a linear increase in genetic differences with geographic distances.

***C. album***: Similar to the UPGMA clustering (**Figures 1**, **2**), the Mantel test indicated no significant isolation-by-distance (IBD) pattern among 17 populations (r = -0.035, P (rxy-rand > = rxy-data) = 0.32) (**Figure 3B**) and among 13 Iranian populations (r = 0.097, P (rxy-rand > = rxy-data) = 0.06) (**Figure 3D**). Similarly, the R^2^ values for the correlation plots of geographical and genetic distances do not support the hypothesis that these two factors are correlated.

## 4 Discussion

The genetic structure analysis we show in **Figures 1**, **2** revealed that the sampled populations of both *A. retroflexus* and *C. album* exhibit a high degree of genetic diversity between the different populations. This conclusion holds true regardless of whether they the analysis only considered the populations sampled from Iran or when geographically isolated populations from Spain or France are included. Analysis of molecular variance results indicate that most of the genetic variation (F_ST_ = 0.71 in *A. retroflexus* L. and 0.7 in *C. album* L.) was found among populations. Additionally, our data indicate that there is little genetic diversity within a given population of *A. retroflexus* or *C. album*. Theory predicts that colonization of new areas will be associated with population bottlenecks that reduce within population genetic diversity and increase genetic differentiation among populations. This should be especially true for weedy *A. retroflexus* and *C. album* (Amsellem et al., 2000). We see a high number of unique alleles in nearly all of the sampled populations (**Table 2**). Together these data are consistent with independent introductions of predominantly inbreeding populations which therefore have naturally low gene flow between the populations. This agrees with previous studies that reported a high genetic diversity among *Amaranthus* populations using RAPD markers (Transue et al., 1994; Mandal and Das, 2002) and other values of genetic differentiation (Aguayo et al., 2013; Ueno et al., 2015) including the average value of F_ST_ for autogamous species using molecular markers which is 0.70 (Nybom and Bartish, 2000). In principle, a high level of genetic diversity provides a varied genetic toolbox that enables adaptation to an extensive range of ecosystems (Dekker, 1997) while self-fertilization can enhanced fitness of weedy populations if the benefits of local adaptation outweigh potential cost of inbreeding (Verhoeven et al., 2011).

The presence of private alleles is important because it may indicate disparate evolutionary paths were taken by the different populations (Yang et al., 2013). Although the presence of these private alleles may be attributed to high mutation rates (Kronholm et al., 2010), it is more likely that as others have concluded (Ueno et al., 2015; Wyman et al., 2019) that the populations faced unique selection pressures after introduction and that they were relatively recently and independently introduced into the locations from which they were sampled. These species each have excellent dispersal abilities (Maurya and Ambasht, 1973; Knezevic and Horak, 1998); and highly diverse morphologies and biochemistries (Hamidzadeh et al., 2021) which we know contributes to a plant’s potential to rapidly and efficiently colonize new habitats. Plant morphology, phenology and breeding system significantly influences genetic diversity where in general, long-lived and outcrossing species have higher levels of genetic diversity than selfing and/or clonal plants (Hamrick and Godt, 1996). Therefore, low genetic diversity within populations is what is expected from these mainly autogamous weedy species (Barrett et al., 2008), since self-fertilization reduces the proportion of heterozygous loci in individuals, causing fixation of homozygous loci (Hamilton, 2009).

The Mantel tests we conducted show isolation-by-distance (IBD) and therefore positive correlations between genetic distances and geographic distances among *A. retroflexus* populations (**Figure 3**). However, the clustering analysis (**Figures 1**, **2**) did not show grouping based on proximity and there was little evidence for gene flow between the populations. We also see persistence of unique alleles among populations. Indeed, other studies have reported similar genetic patterns for plants with self-reproduction (Atwater et al., 2018), clonal growth (Li and Dong, 2009), fast-growth (Barluenga et al., 2011) and high-density populations (Vekemans and Hardy, 2004). This was not the case with the *C. album* populations where the Mantel test suggested that the distribution of genetic diversity among *C. album* populations is not explained by geographical distances as we found no evidence of isolation by distance among the locations sampled. Although our small sample could influence our ability to accurately conclude a relationship between geographic and genetic distances, Guggisberg et al. (2012) similarly concluded that colonization of Canada thistle (*Cirsium arvense*) was the result of independent and multiple introductions because of data showing their populations exhibited different genetic fingerprints and lacked a correlation between genetic and geographic distances. *C. album* populations are most commonly found on disturbed areas (CABI, 2020), and therefore dispersal driven by human activity is likely in these species (Krak et al., 2019). As a result, our lack of correlation between genetic and geographic distances of populations implies that seed dispersal mechanisms and colonization history have influenced the spatial distribution and genetic diversity we observed, similarly to other species (Heywood et al., 2007).

Although it is well accepted that European *A. retroflexus* is a neophyte (Axmanová et al., 2021), neither the precise origin nor the first report of *C. album* L. are precisely known (CABI, 2020). Linnaeus described the species in 1753 (Rickett, 1958, Flora Europaea: *C. album*), as inhabiting most of Europe. Plants thought to be native to Eastern Asia are included under *C. album*, but often differ from European specimens (Zhu et al., 2003). In extent at the beginning of the period, *C. album* is domesticated in the Himalayan region where it is grown as a grain crop. There is archaeological evidence to suggest it was cultivated as a pseudo-cereal in Europe in prehistory (Stokes and Rowley-Conwy, 2002). Historical range aside, these references showed that *C. album* cannot be considered native to Iran (Kazi et al., 2007; Ghorbani et al., 2010; Hassannejad et al., 2014). According to A. Pahlevni (pers.comm.), there is no evidence of historical gatherings of this weed from Iran. Further details of the native ranges and known history of global distribution patterns for these two species are given in Hamidzadeh et al. (2021).

Quantitative data about the spatial distribution of genetic diversity is essential to better understand the relationships between life-history traits, stochastic effects, gene flow, selection pressures and environmental factors (Escudero et al., 2003). The genetic diversity analyses we have conducted here using ISSR molecular markers revealed that the studied populations of weedy *A. retroflexus* L. and *C. album* L. have low intra-population genetic diversity and are divergent among each other. Combining genetic variation, gene flow, population genetic structure and IBD analysis, suggest that the existing genetic variation and spatial genetic structure of populations were caused by distinct introduction events of these species to these locations. Self-fertilization, drift events, colonization by few individuals, different selection pressures acting even within small geographic areas may have influenced the genetic diversity of these populations. Although these results are limited to selected populations from Iran with French and Spanish outgroups, it is useful for understanding the weediness of *A. retroflexus* and *C. album* into Iran and can be extended to further noxious populations covering a wider geographic distribution.

## 5 Conclusion

Analysis of ISSR markers in this set of *A. retroflexus* L. and *C. album* L. populations allowed us to assess the effects of geographic distance on population structure as it was extremely unlikely that genetic exchange would have occurred naturally between Iranian and French or Spanish populations. UPGMA clustering of ISSR data support our hypotheses showing that (1) it is likely the Iranian, French and Spanish populations of *A. retroflexus* L. and *C. album* L. were established by individuals from multiple different sources and (2) isolation-by-distance (IBD) has occurred particularly in *A. retroflexus* L. where the likelihood of gene flow is inversely related to distance. However, we show no evidence of isolation by distance among the *C. album* L. populations, indicating geographic distance or geographic barriers may not be the only factor affecting gene flow. Our results show genetic diversity between populations of *A. retroflexus* L. and *C. album* L., which may help explain their diverse phenotypic and biochemical traits and help to explain their success as noxious weeds. Our data supports the theory that in both species, the populations we have sampled have been genetically isolated and multiple introduction events occurred giving rise to these weedy populations.

Knowledge about genetic relatedness within and between populations is crucial for understanding how the populations came to be established as well as for designing successful weed management schemes to deal with them. Herein we evaluate the genetic diversity of Iranian, French and Spanish populations of *A. retroflexus* L. and *C. album* L. using ISSR primers. We were able to obtain an efficient and effective assessment of genetic diversity in *A. retroflexus* L. and *C. album* L. populations. While a large number of molecular markers (dominant and co-dominant) would have improved our analyses as would increased sample sizes or ranges, the amplification of many polymorphic loci indicated the set of ISSR primers we used was sufficient to assess the genetic diversity among the existing populations. Here, we demonstrate that ‘weedy’ traits, such as selfing and clonal growth may result in populations that have distinct phenotypic and genetic fingerprints depending on the selecting conditions. The low genetic variation within populations and maladapted gene flow among populations seen in our results indicates that every population is a unique, evolutionarily-significant unit and should be considered as an independent management unit for weed population control.

## Data availability statement

The original contributions presented in the study are included in the article/**Supplementary Material**. Further inquiries can be directed to the corresponding author.

## Author contributions

SH performed the experiments, data collection, data analysis, figure preparation, and writing of the manuscript. MA conceived the original data, formulated the research plan, oversaw the research, and writing of the manuscript. MM and DM contributed to data analysis and writing of the manuscript. All authors contributed to the article and approved the submitted version.

## Supplementary material

Supplementary Figure 1 Maps showing the sample collection locations for ecotypes of *A. retroflexus* (**A**, top map with pink pins) and *C. album* (**B**, bottom map with yellow pins). The samples were also described in Hamidzadeh Moghadam et al., 2021). The black bar in the Caspian Sea is showing 200 km. For *A. retroflexus*, the three collections from Spain all fall under the same pin location on this scale. Likewise, for *C. album*, the two Spanish and the two French ecotypes are represented by the same pins.

Supplementary Figure 2 ISSR patterns generated by UBC810 primer on 16 *A. retroflexus* **(A)** and 17 C*. album* **(B)** populations DNA. The ladder is a 50 bp DNA Ladder (SinaClon). Lanes designate based on.
