# Supplementary figures and images for: Genetic variation of Amaranthus retroflexus L. and Chenopodium album L. (Amaranthaceae) suggests multiple independent introductions into Iran
Source: Front Plant Sci. 2023 Jan 4;13:1024555. doi: 10.3389/fpls.2022.1024555 (PMC9847890; doi:10.3389/fpls.2022.1024555)

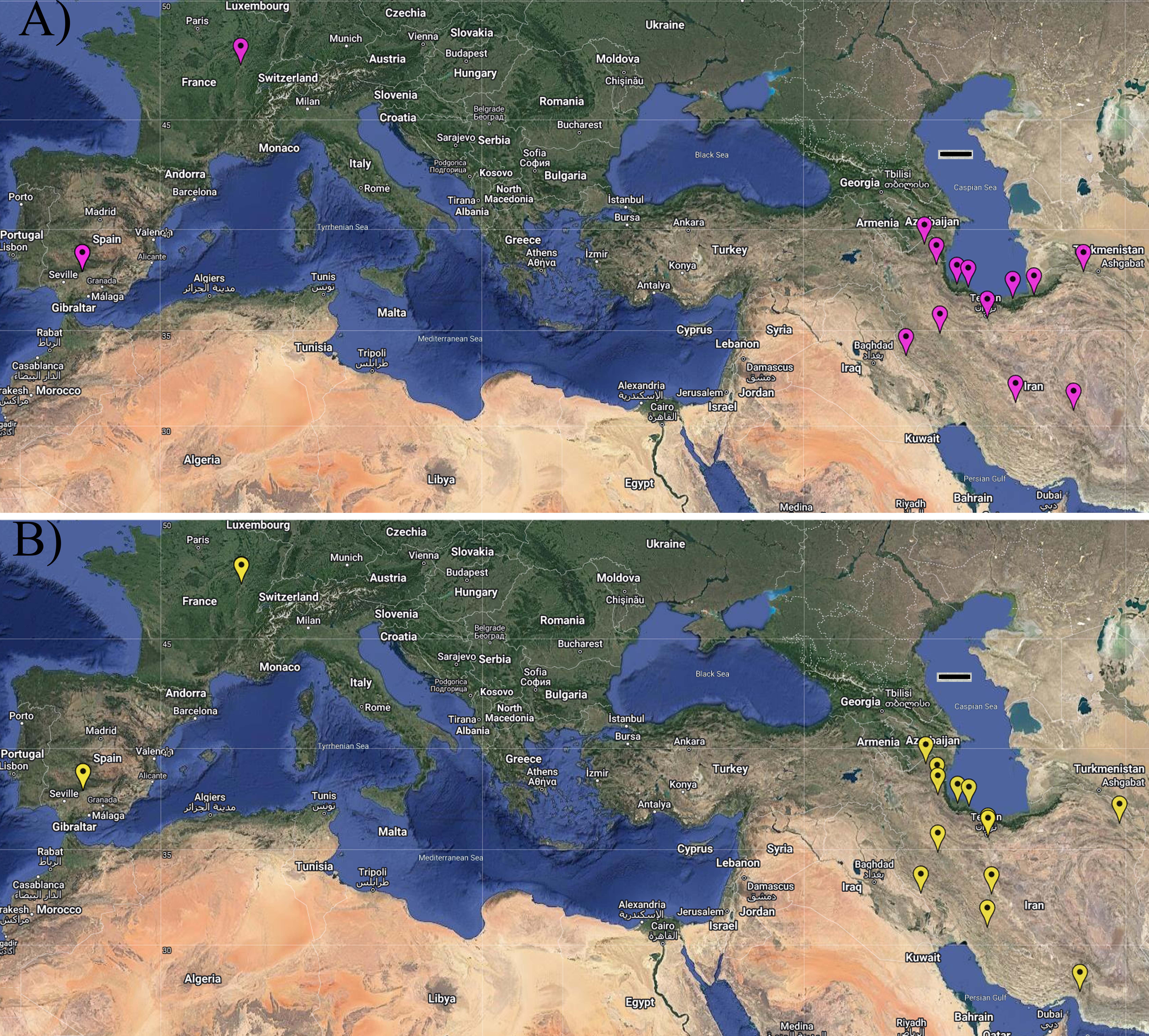

Supplement: Supplementary Figure 1 — Maps showing the sample collection locations for ecotypes of A. retroflexus (A, top map with pink pins) and C. album (B, bottom map with yellow pins). The samples were also described in Hamidzadeh Moghadam et al., 2021). The black bar in the Caspian Sea is showing 200 km. For A. retroflexus, the three collections from Spain all fall under the same pin location on this scale. Likewise, for C. album, the two Spanish and the two French ecotypes are represented by the same pins. [file Image_1.jpeg]

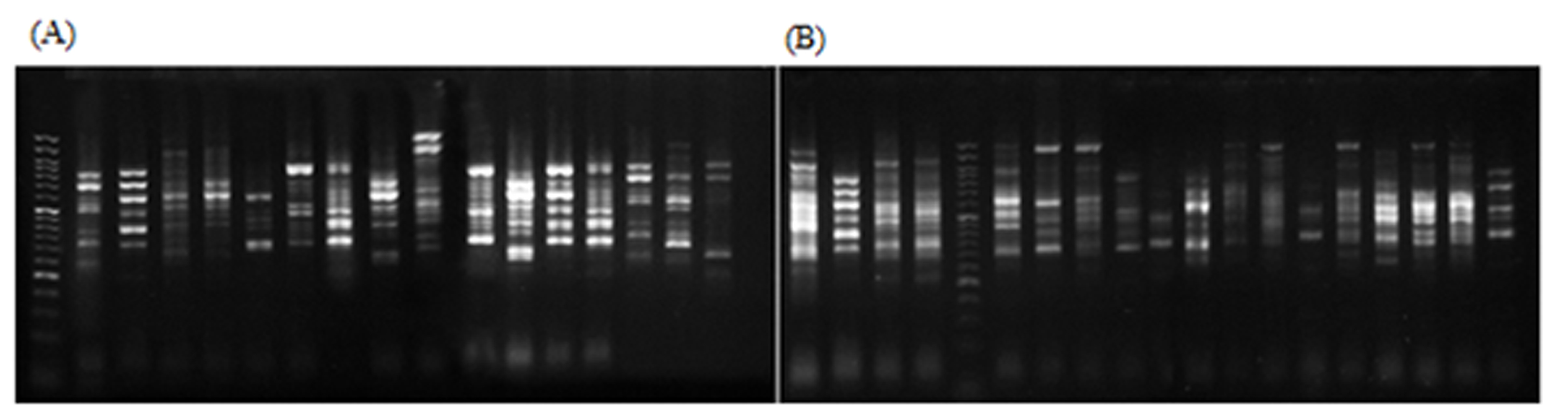

Supplement: Supplementary Figure 2 — ISSR patterns generated by UBC810 primer on 16 A. retroflexus (A) and 17 C. album (B) populations DNA. The ladder is a 50 bp DNA Ladder (SinaClon). Lanes designate based on. [file Image_2.jpeg]
